# The Beneficial Effects of Hesperidin and Exercise on the Histology and Biochemical Parameters of Surgically Induced Endometriosis in a Rabbit Model

**DOI:** 10.5935/1518-0557.20250183

**Published:** 2026

**Authors:** Idowu Sunday Oyeleye, Barakat Olamide Ishola, Busuyi Akinola, Abiola Hannah Oduntan, Victor Okoliko Ukwenya, Ganiyu Oboh

**Affiliations:** 1 Department of Biomedical Technology, Federal University of Technology, Akure Nigeria P.M.B., 704, Akure 340001, Nigeria; 2 Department of Human Anatomy, Federal University of Technology, Akure. Nigeria P.M.B., 704, Akure 340001, Nigeria; 3 Department of Biochemistry, Federal University of Technology, Akure. Nigeria P.M.B., 704, Akure 340001, Nigeria

**Keywords:** Uterus, citrus fruits, flavonoids, antioxidants, aerobic exercise

## Abstract

**Objective:**

Endometriosis is a gynecological disorder marked by the formation of endometrial tissue (gland and stroma) outside the uterine cavity. Macrophages, erythrocytes, and apoptotic endometrial tissue transplant into the peritoneal cavity, free radicals, and oxidative stress play a vital role in endometriosis. This study focused on unraveling the combinatorial effect of treadmill exercise with hesperidin (HESP, a citrus flavonoid) on histoarchitecture and biochemical [reactive oxygen species (ROS), nitric oxide (NO), total thiol (T-SH), glutathione (GSH), TBARS levels, catalase, glutathione peroxidase (GPx), superoxide dismutase (SOD), glutathione-s-transferase (GST), and monoamine oxidase activities] molecules in endometrial tissue of female rabbits.

**Methods:**

The rabbits underwent surgery by resecting one uterine horn, isolating the endometrium, and fixing the tissue segment to the pelvic peritoneum, and they were distributed into 5 groups (n = 6). Group 1: normal control (NC) Group 2: untreated endometriotic rabbits (ENDO); Group 3: HESP-treated endometriotic rabbits (eNDO + HESP); Group 4: exercised endometriotic rabbits (ENDO + EXER); Group 5: exercised endometriotic rabbits administered with HESP (ENDO + HESP + EXER).

**Results:**

The results revealed that antioxidant enzyme activities and non-antioxidant molecules were reduced in untreated endometriotic rabbits compared to NC. However, there was a significant increase in antioxidant status in exercised endometriotic rabbits treated with hesperidin.

**Conclusions:**

This finding revealed that combining physical exercise with the consumption of hesperidin-rich fruits can be explored to alleviate oxidative stress, a major risk factor in endometriosis.

## INTRODUCTION

Endometriosis is a benign estrogen-dependent gynecological disorder in which endometrial tissue grows outside the uterus. Endometriosis affects about 2 to 22% of reproductive-age women; and 40 to 60% of women who have painful menstruation, as well as 25 to 50% of infertile women (Yang *et al.,* 2004). Despite its well-established pathophysiology, substantial morbidity and healthcare expenditures are associated with endometriosis, and the specific reason is largely unclear (Viganò *et al.,* 2004). However, oxidative stress - an imbalance between the radical species and endogenous antioxidant molecules has been fingered as a culprit in the pathogenesis of endometriosis, resulting in a peritoneal cavity inflammatory response (Augoulea *et al.,* 2009).

Reactive oxygen species (ROS) are inflammatory mediators that control cell growth and elicit harmful consequences during normal oxygen metabolism (Jena *et al.,* 2023). Cells have developed various antioxidant systems to limit ROS production and cell damage, including up-regulating antioxidant molecules such as superoxide dismutase, catalase, and glutathione peroxidase activities (Jena *et al.,* 2023). However, oxidative stress occurs when the balance between ROS production and antioxidant defense is disrupted (Lousse *et al.,* 2012). This is in line with the fact that macrophages, erythrocytes, and apoptotic endometrial tissue that are transplanted into the peritoneal cavity by retrograde menstruation are recognized inducers of oxidative stress; hence, peritoneal generation of ROS is involved in the endometriotic condition (Lousse *et al.,* 2012).

Severe endometriosis distorts pelvic anatomy, lowers fertility, and causes changes to the eutopic endometrium, implying that the endometriotic implants communicate with the native endometrium in some way (Rocha *et al.,* 2023). The influence of specific anomalies in the eutopic endometrium of women with endometriosis on fertility, particularly in moderate illness, is now the focus of exciting news in endometriosis research. Changes in the endometrium of women also contribute to the disease’s pathophysiology and proclivity to deteriorate or recur. The condition manifests itself in a variety of ways, from acyclic pelvic discomfort to infertility (Rocha *et al.,* 2023).

Due to the toxicity of synthetic antioxidants used in medicines, natural antioxidants derived from plant species, such as flavonoids, have recently received a lot of attention (Juntachote & Berghofer, 2005) due to their ability to halt free radical production (Ahmadi & Shadboorestan, 2016). Hesperidin is a polyphenolic chemical that occurs naturally in fruits and vegetables. Citrus fruits principal flavonoid, hesperidin, may be extracted in significant concentrations from the rinds of several citrus species (Wilmsen *et al.,* 2005). Hesperidin’s antioxidative properties include substantial reducing power, metal chelating, and radical (hydrogen peroxide, superoxide, and hydroxyl) scavenging abilities (Wilmsen *et al.,* 2005).

On the other hand, physical exercise is known for its positive effects on human antioxidant defenses (Bouzid *et al.,* 2018). Engaging in moderate exercise in an active lifestyle has been shown to help alleviate oxidative stress (Baltaci *et al.,* 2016). The beneficial effects of exercise include releasing myokines, cytokines, interleukins, and other peptides to prevent inflammatory disorders like endometriosis (Golbidi *et al.,* 2012). The prevalence of endometriosis has caused infertility and painful menstrual periods in reproductive women; thereby having a significant impact on their physical, mental, and social well-being. To date, there is no established cure for endometriosis, and most current medical treatments are not suitable for long-term use due to their side-effects. Therefore, this study aims to evaluate the effect of physical exercise with/without hesperidin consumption on the endometriosis-induced oxidative stress markers in female rabbits.

## MATERIALS AND METHODS

### Animal Handling

The study used adult female New Zealand rabbits of size 1.5 – 2.0 kg maintained under regulated conditions of ambient humidity, temperature, and light for 14 days, receiving water and food *ad libitum.* The university’s Ethics Committee approved all animal handling procedures.

### Experimental Procedure

The rabbits were subjected to a running program for 4 days to become familiarized with the treadmills under constant supervision (Such *et al.,* 2008), and those that did not adequately run on the treadmill were excluded from the study. Thereafter, under sterile and strict antisepsis conditions in the laboratory, the animal’s abdominal fur was carefully shaved to get a clean cut of the abdominal skin. The anesthetic agent (35 mg/kg of ketamine and 2 mg/kg diazepam) was administered. The pelvic cavity was opened by a median longitudinal incision of approximately 4 cm at a distance of 4 cm from the pubis. A segment of about 2 cm of the uterine horn was resected, and the horn was closed with Vicryl 6.0. The resected uterine portion was immersed inside normal saline to avoid desiccation of the tissue and then cut longitudinally to obtain a 10 by 10 mm fragment from the tissue. The fragment was sutured to the peritoneal wall at the junction where two blood vessels meet, and the endometrial tissue was faced directly to the abdominal cavity using Vicryl 6.0 suture. The abdominal incision was then closed layer by layer using Vicryl 2.0 suture. Diclofenac injection was given to the animal after surgery to relieve pain (Rosa-e-Silva *et al.,* 2010).

### Animal Grouping and Experimental Design

The rabbits were grouped into five groups (n=6) as follows: Group 1 - Normal control (NC); Group 2 - untreated endometriotic rabbits (ENDO); Group 3 - endometriotic rabbit treated with hesperidin (ENDO + HESP); Group 4 – physically exercised endometriotic rabbit (ENDO + EXER); Group 5 - physically exercised endometriotic rabbit treated with hesperidin (ENDO + HESP+ EXER). The animals were allowed to recover from surgery for 5 days. Hesperidin solution was prepared and orally administered to group 3 (ENDO + HESP) and 5 (ENDO + HESP + EXER) rabbits at a dose of 50 mg/kg every day for 2 weeks (Melekoglu *et al.,* 2018). The animals in groups 4 (ENDO + EXER) and 5 (ENDO + HESP + EXER) were placed on a treadmill for two weeks. The treadmill was set at a speed of 1.2 m/s for the first day, and the rabbits were placed on it for 10 min. The treadmill’s speed and time for exercise were later increased to 2m/s for 20 min.

### Animal Slaughter, Tissue Collection, and Preparation for Biochemical Assays

The hesperidin administration and physical exercise protocols were done concurrently for two weeks. Thereafter, the animals were anaesthetized with ketamine, and cervical dislocation was done. Grafted endometriosis tissue. The endometrial-implanted tissue in the rabbits was collected and separately homogenized in 0.1M phosphate buffer (pH 7.4) in a laboratory homogenizer. The homogenate was centrifuged, and the supernatant was used to estimate biochemical assays.

### Hematoxylin and Eosin Stain of Endometriotic Tissue

The harvested tissues were fixed in Bouin’s fluid for histological analysis. The uterus of the control and the experimental groups as well as the ectopic surgically grafted endometrial tissue together with portions of the peritoneal walls were also harvested for analysis. The fixed tissue was dehydrated with alcohol, cleared with xylene, and embedded in paraffin wax. The tissue was cut to produce 4–5 µm sections using a microtome, fixed on the slides, stained with hematoxylin and eosin (H and E), and then viewed under a light microscope (Olympus/3H -Tokyo, Japan).

### Biochemical Assays

#### Determination of reactive oxygen species (ROS) level

ROS level was estimated as equivalent to H_2_O_2_, according to the method reported by Oboh *et al.* (2018) using the reagent n-n-diethyl-para-phenylenediamine (DEPPD).

#### Determination of thiobarbituric reactive acid species (TBARS)

TBARS levels in the endometrial tissue homogenates of experimental rabbits were carried out using the described method by Ohkawa *et al.* (1979).

#### Nitric oxide (NO) assay

The formed nitrous acid diazotises sulphanilamide, and the product, coupled with N-(1 1-naphthyl) ethylenediamine, was determined in the acid medium and the presence of nitrate. The resulting azo dye, which has a bright reddish-purple colour, was measured at 570 nm.

#### Determination of catalase activity

This assay was carried out using Sinha’s method (Sinha, 1972) with a dichromate (acetic acid) solution and measured at 620 nm for 3 min at 30-second intervals.

#### Determination of superoxide dismutase (SOD) activity

The determination of SOD activity in the uterus was based on the inhibition of the radical superoxide reaction with adrenalin, as described by Misra & Fridovich (1972).

#### Determination of glutathione peroxidase (GPx) and glutathione transferase (GST) activities

Glutathione peroxidase was determined by the method described by Rotruck *et al.* (1973) using Ellman reagents, while GST was carried out using 30 mM GSH as described by Mannervik and Guthenberg method (Mannervik & Guthenberg, 1981).

#### Determination of total glutathione (GSH) content

Total glutathione (GSH) was determined using Ellman’s method (Ellman, 1959).

#### Determination of monoamine oxidase (MAO) inhibition assay

The MAO activity was measured using the modified method of Ademosun and Oboh (Ademosun & Oboh, 2014).

### Data Analysis

All data were expressed as mean ± standard error of the mean (SEM). One-way analysis of variance (ANOVA) was used to analyze the differences between the groups with the aid of GraphPad Prism 5.0 Software (GraphPad Software Inc., San Diego, CA). Followed by the post hoc Tukey’s test, *p*<0.05 represented a significant difference in both analyses.

## RESULTS

**Figure 1:**
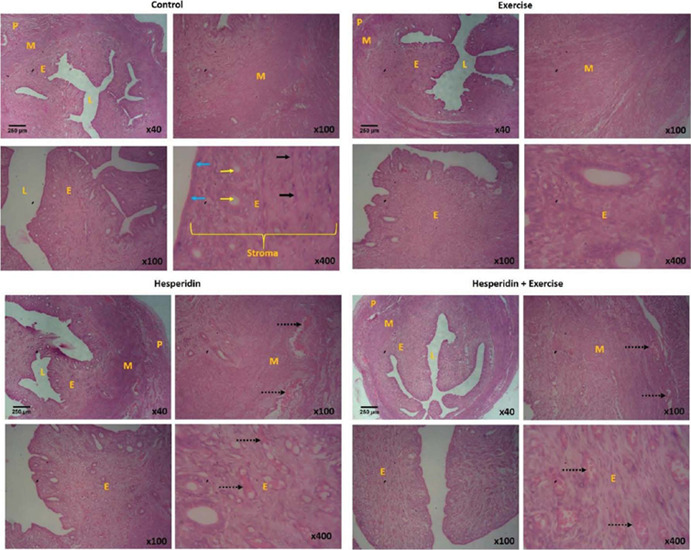
Shows the uterine histology of the control group (H&E; x40, x100, x400). M -myometrium; P - perimetrium; E - endometrium; L – _lumen; Blue arrows – _surface epithelium of the endometrium; Yellow arrow – _endometrial glands; Black arrows – _nuclei of endometrial stroma cells; Black dotted arrows – _hemorrhage.

**Figure 2:**
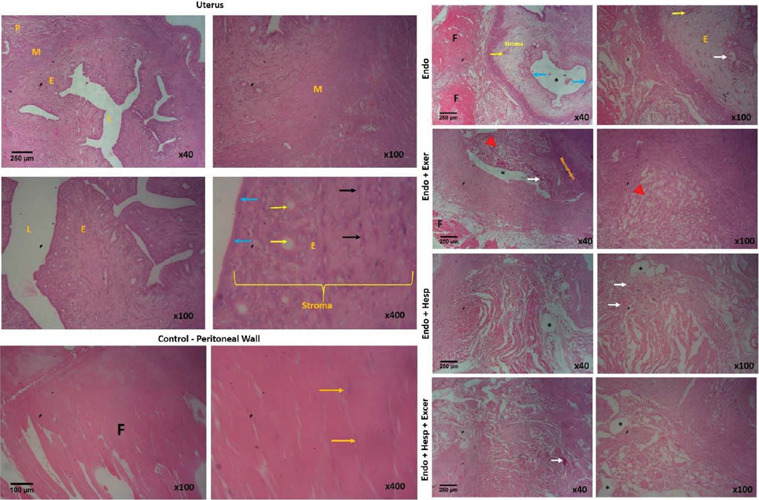
Shows the histology of the uterus and peritoneal wall of the control groups (H&E; x40, x100, x400) and the endometriotic tissue of the experimental groups (H&E; x40, x100). M - myometrium; P - perimetrium; E - endometrium; L - _lumen; F - Fasciculi; Long yellow arrows – _nuclei of muscle cells; Blue arrows – _surface epithelium of the endometrium; Short yellow arrows – _endometrial glands; Black arrows – _nuclei of endometrial stroma cells. White arrows – _hemorrhage/artery; *represents endometrial cyst; Red arrowhead - fibro-adipose tissue.

The Normal control group showed the typical uterine histology. There are three distinct layers: the endometrium, myometrium, and perimetrium. The endometrium’s surface epithelium is pseudo-columnar and encloses a centrally placed lumen. The underlying stroma is cellular and composed of ovoid cells with scanty cytoplasm. The endometrial stroma has numerous endometrial glands dispersed within it and is well demarcated from the myometrium, as evidenced by the fascicular nature of the myometrium. The myometrium comprises smooth muscle cells containing one or two elongated eosinophilic nuclei. Surrounding the myometrium is a serous layer known as the perimetrium (Fig. 1). The peritoneal wall of the normal control group was sectioned for the purpose of comparison to the ectopic endometrial tissues, and it was found to be characterized by parallel longitudinal fibers with myocytes containing one or two elongated nuclei (Fig. 2). The Endometriosis group presents the histological characteristics of endometriosis defined by the presence of glands, small arteries, endometrial cysts, endometrial stroma, and endometrial epithelium (Fig. 2). The animal placed on the exercise only possessed cystic dilatations of the endometrial glands, which arise from intra-glandular hemorrhage. Endometrial glands, fibro-adipose tissue, and stroma are also present centrally. Peripheral to these, there are numerous surrounding macrophages (Fig. 2). The hesperidin, as well as hesperidin plus exercise groups, present endometriosis histology characterized by obvious endometrial glands, hemorrhage, and endometrial cysts (Fig. 2). The endometriosis in exercise only, hesperidin, and hesperidin plus exercise groups are poorly formed compared to that in the untreated endometriosis group.

**Figure 3:**
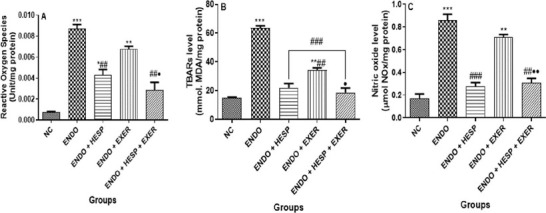
a. Reactive oxygen species (ROS), b. Thiobarbituric acid reactive species and c. nitric acid (NO) levels in the homogenate of the uterus tissue of an endometriotic rabbit (n = 6). Values represent mean standard deviation (SD) and are statistically different at: *p<0.05, **p<0.01, ***p<0.001 *vs.* normal control; ^#^*p*<0.05, ^##^*p*<0.01, ^###^*p*<0.001 *vs.* Endo; **p<0.05 *vs.* ENDO + EXER. Key: ENDO = Endometriosis; HESP = Hesperidin; Excer = Exercise.

An increased level of ROS was observed in the untreated ENDO group. Subsequently, the animal was treated with HESP and EXER, and there was a decrease in ROS level. However, those treated with HESP+EXER therapy exhibited a remarkably low level of ROS at par with that of NC (Fig. 3A). TBAR levels were significantly higher in the untreated ENDO group than in the NC group. Meanwhile, the group treated with HESP or EXER had little or no difference in the TBAR level. ENDO rats treated with combined HESP plus EXER had a close value level of TBAR (*p*>0.05) to the NC’s TBAR level (Fig. 3B). NO levels were significantly increased in the ENDO group compared to the NC group. The ENDO rabbit treated with EXER had a reduced NO level, while the treatment with HESP only possessed a reduced NO value relative to NC. The HESP+EXER group indicated a suitable level of NO with respect to NC (Fig. 3C).

**Figure 4:**
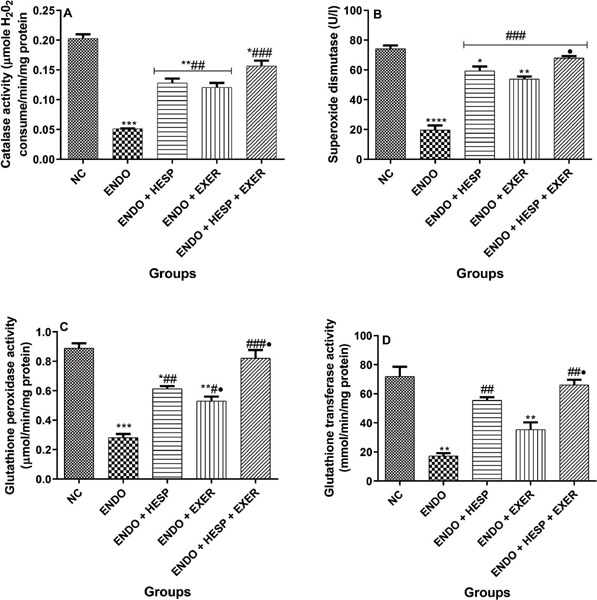
a. Catalase, b. Superoxide dismutase (SOD); c. glutathione peroxidase (GPx) and d glutathione–S-Transferase (GST) activities in the homogenate of uterus tissue of endometriotic rabbit (n = 6). Values represent mean standard deviation (SD) and are statistically different at: *p<0.05, **p<0.01, ***p<0.001 *vs.* normal control; *p<0.05, ^##^*p*<0.01, ^###^*p*<0.001 vs. Endo; **p<0.05 *vs.* ENDO + EXER. Key: ENDO = Endometriosis; HESP = Hesperidin; Excer = Exercise.

The ENDO condition reduced catalase activity, but an extended treatment in another group with HESP and EXER described an improved catalase status (Fig. 4A). This attribute can be due to the presence of HESP and EXER. Though treatment with HESP had no significant difference from EXER treatment, the combined HESP+EXER therapy possesses the greatest increase in catalase activity. SOD activity, as shown in Fig. 4B in a combined treatment of HESP+EXER, exhibited a significant increase that was greater than even the NC. The group treated with EXER alone showed an improved SOD activity, significantly higher than the group treated with HESP alone. The ENDO condition decreased GPx activity (Fig. 4C), but the treatment with HESP and EXER improved GPx activity. Perhaps treatment with EXER has no significant difference compared to HESP treatment, but the combined use of HESP+EXER therapy indicated the most suitable level of GPx activity. The ENDO condition reduced the activity of glutathione–s-transferase (Fig. 4D) relative to NC, but the treatment group with HESP and EXER exhibited improved GST activity. The treatment with HESP is slightly higher than that with EXER, but the combined treatment of HESP+EXER showed the most increased GST activity, almost at the same level as NC.

**Figure 5:**
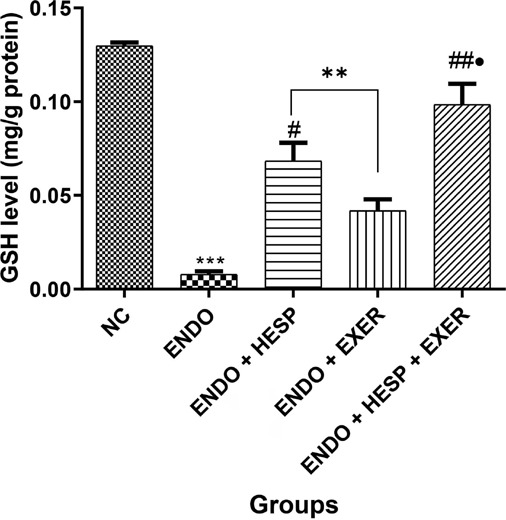
Glutathione (GSH) level in an endometriotic rabbit’s homogenate of the uterus tissue (n = 6). Values represent mean standard deviation (SD) and are statistically different at: *p<0.05, **p<0.01 *vs.* normal control; *p<0.05, ^##^*p*<0.01 *vs.* Endo; *p<0.05 vs. ENDO + EXER Key: ENDO = Endometriosis; HESP = Hesperidin; Excer = Exercise.

**Figure 6:**
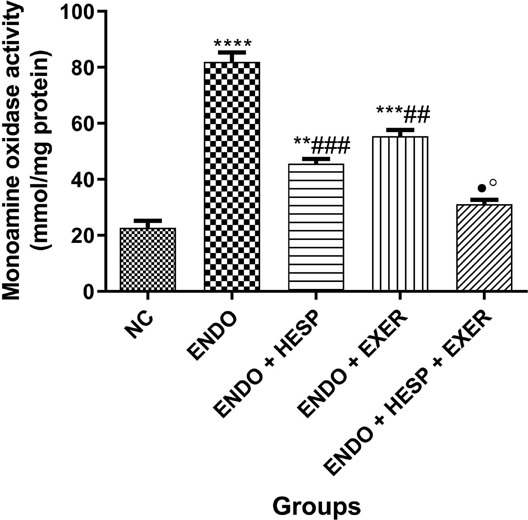
Monoamine oxidase activity in the homogenate of the endometriotic rabbit’s uterus tissue (n = 6). Values represent mean standard deviation (SD) and are statistically different at: *p<0.05, ***p<0.01 *vs.* normal control; ^##^*p*<0.01, ^###^*p*<0.001 *vs.* Endo; *p<0.05 *vs.* ENDO + HESP, º*p*<0.05 *vs.* ENDO + EXER. Key: ENDO = Endometriosis; HESP = Hesperidin; Excer = Exercise.

The combined effect of HESP+EXER as a therapy for ENDO tissue on GSH level is presented in Figure 5, showing a significant increase in GSH level. The group treated with HESP alone also showed an improved level of GSH compared with the group treated with EXER alone, which had a lower GSH level. The ENDO group exhibited a reduced level of GSH. The result of monoamine oxidase (MAO) activity in endometriosis tissue (lesion) shows that there was a significant increase (Fig. 6) of MAO activity. MAO activity was high in the untreated ENDO group. However, there is a close-range activity of MAO between the ENDO + HESP group and the ENDO + EXER group. There is a drastic decrease in MAO activity in the group treated with HESP + EXER (Fig. 1).

## DISCUSSION

The endometriosis medical therapy options available for clinical use are ineffective and have significant side effects. No convincing evidence suggests one treatment over another (Dunselman *et al.,* 2014). Natural antioxidants and effective therapies for endometriosis are currently being researched. Due to oxidative stress involvement in the pathogenesis of endometriosis, effective antioxidant therapies with little or no side effects are being developed (Melekoglu *et al.,* 2018). This study investigates the effect of hesperidin and physical exercise on a rabbit model of surgically induced endometriosis.

Significant histological changes were observed in the endometriotic lesions. Destruction of the endometrium was more evident in the columnar epithelial layer and glands. Prominent hemorrhage, vascular congestion, necrosis, inflammatory cell infiltration, cystically dilated glands, and significant blood cell accumulation in the lumen of endometrial lesions were detected in the endometrial lesion layers. All histological parameters were improved after hesperidin and exercise administration (Fig. 1).

ROS are byproducts of normal cellular metabolism that play important roles in signaling pathways, including intracellular signal transmission, metabolism, proliferation, and apoptosis. ROS are formed over time in response to long-term environmental stress, which can cause considerable damage to cell structure and function (Fleury *et al.,* 2002). This biological reduction of molecular oxygen is a source of reactive oxygen species (ROS), including the primary free radical superoxide anion (O_2_^•_^) and the major non-free radical hydrogen peroxide (H_2_O_2_) (Zorov *et al.,* 2014). The result showed that ROS was prominent in untreated ENDO rabbits, but the animals treated with hesperidin revealed a low level of ROS. The combined therapy of HESP+EXER significantly decreases ROS level (Fig. 2). This is consistent with the study of Liu *et al.* (2017), which revealed that HESP could block the production of free radicals.

Uptake of oxygen, generation of lipid radicals, and rearrangement of double bonds in unsaturated and polyunsaturated fatty acids are all part of the lipid peroxidation process (Dasgupta & Klein, 2014). TBARS is a lipid peroxidation end product, malondialdehyde, a reactive aldehyde formed by lipid peroxidation of polyunsaturated fatty acids. This study revealed that TBARS was high in the tissue of untreated ENDO rabbits. However, the combined therapy of exercise and hesperidin alleviates TBARS level (Fig. 3B). This is in line with the study of Homayouni *et al.* (2017), which showed that hesperidin supplement alleviates oxidative DNA damage and lipid peroxidation.

Nitric oxide (NO) is a vasodilator produced by the nitric oxide synthase (NOS) enzyme, from **L**-arginine (Najafi *et al.,* 2012), but when combined with superoxide (O_2_), it produces peroxynitrite, a hazardous radical with adenomyosis, a form of endometriosis that affects the myometrium (Jena *et al.,* 2023). High levels of NO negatively influence fertility in several ways: toxic embryos, inhibit implantation, and affect contractile activity in the oviduct (Osborn *et al.,* 2002). Our research showed that the untreated endometriosis group’s NO level was high. Still, treatment with HESP and/or exercise showed a complementary decrease.

Still, it was pronounced in the combined treatment of hesperidin and exercise (Fig. 3C). Previous studies indicated that hesperidin attenuated NO production in mice macrophage cell lines (Sakata *et al.,* 2003) due to its antioxidant, anti-inflammatory, anti-proliferative, and anti-carcinogenic properties (Parhiz *et al.,* 2014). In this study, both physical exercise and hesperidin exhibited antioxidative potentials.

The antioxidant enzyme catalase (CAT) is found in practically all biological tissues, whereby its breakdown of hydrogen peroxide to water and molecular oxygen uses either iron or manganese as a cofactor, thereby completing the detoxification process (Ighodaro & Akinloye, 2018). From Fig. 4A, the catalase activity in the endometriosis group was significantly lower compared to NC, thus indicating oxidative stress (Sourial *et al.,* 2014). The combined treatment of hesperidin and exercise increased catalase activity relative to the untreated endometriosis group. Antioxidant supplements such as hesperidin induce maturity and growth of follicles by lowering oxidative stress, activating enzymes such as CAT, promoting the development of follicles and embryonal development, and improving fertilization rate; thereby preventing reproductive diseases such as unexplained infertility and endometriosis (Wang *et al.,* 2017).

SOD is an important endogenous antioxidant enzyme that acts as a first line of defense against free radical species (Perhiz *et al.,* 2014). The present study indicates that aerobic exercise is a good modulator of SOD activity in the body. However, its combination with hesperidin raises the enzyme activity above normal control (Fig. 4B). The study by Yan & Spaulding (2020) found that endurance (aerobic) exercise increased the SOD activity in the aorta of mice and human plasma. Exercise training increases the amount of SOD protein in peripheral organs such as the heart, kidneys, liver, heart, lung, and adipose tissue. These data support the hypothesis that exercise increases SOD expression in skeletal muscle, the body’s biggest organ, resulting in increased extracellular antioxidant defense in the circulation and peripheral tissues as a molecular transducer of the benefits of exercise to health and disease (Yang & Spaulding, 2020).

Glutathione peroxidase (GPx) is an intracellular enzyme that breaks down hydrogen peroxide to water and lipid peroxides to their corresponding alcohols (Gill & Tuteja, 2010). GPx is particularly important in suppressing lipid peroxidation; thereby protecting cells from oxidative stress (Gill & Tuteja, 2010). From Fig 3C of the result, the GPx activity was greatly reduced in the endometriosis group compared to the NC group. However, the combined treatment of HESP+EXCER significantly increased GPx activity. Physical activity improves antioxidant defenses and lowers lipid peroxidation levels in adults and aged individuals. The exercise-induced ROS generation results in increased activity of enzymatic antioxidants, which then leads to increased resistance to oxidative challenges, including a wide variety of oxidative stress-related diseases such as diabetes, mitochondrial myopathies, and endometriosis, among others (Baltaci *et al.,* 2016).

Glutathione–s-transferase (GST) is a subfamily of phase II detoxification enzymes that uses GSH as a co-factor to derivatize cellular electrophiles of various origins, such as xenobiotics and endogenous reactive metabolites; thereby providing yet another important mechanism of cell protection against harmful electrophiles (Scirè *et al.,* 2019). The results from Fig. 4D showed an increase in GST activity in animals with endometriosis treated with hesperidin and the combination of hesperidin plus exercise, yielding a higher level of enzyme activity. In the study of Melekoglu *et al.* (2018), oxidative stress markers were significantly higher in the endometriosis group, and increased antioxidant activity was observed in the endometriotic foci of rats treated with hesperidin and nerolidol.

Glutathione (GSH) is a co-substrate of GPx, which allows peroxides (hydrogen and lipid peroxides) to be reduced and GSSG to be produced. NADPH-reducing equivalents and glutathione disulfide reductase catalysis are then used to convert GSSG to 2GSH (Gaucher *et al.,* 2018). The present study revealed that the GSH level was significantly low in the untreated ENDO group, and the combined therapy of HESP+EXCER improved GSH level. Scutiero *et al.* (2017) reported the role of oxidative stress in the development and progression of endometriosis; and therapeutic approaches involving flavonoids to prevent the formation of endometriosis. Thiols are the most abundant antioxidants in the body, accounting for most total antioxidants, and playing an important role in the defense against radical species (Prakash *et al.,* 2009). Glutathione is made up of intracellular and extracellular thiols that are either free (oxidized or reduced glutathione) or attached to proteins (Prakash *et al.,* 2009). Aside from its involvement in free radical defense, GSH is involved in detoxification, signal transduction, apoptosis, and a variety of other molecular processes. The increased level of GSH reduced the risk of endometriosis and infertility (Prakash *et al.,* 2009). The result from Figure 5 proves that endometriosis reduces the expression of GSH, but it gets improved when treated with hesperidin or exercise, and a significant increment when treated with the combination of exercise and hesperidin.

The monoamine oxidase (MAO), which is found in the mitochondrial membrane, has been demonstrated to be a key cause of oxidative stress (Adefegha *et al.,* 2021). It uses flavin adenine dinucleotide as a cofactor to catalyze the oxidative deamination of a range of monoamines (Henriquez *et al.,* 2006). Untreated endometriosis group expressed higher levels of MAO, therefore increasing the chances of infertility. However, induced animal groups placed on exercise showed a decrease in the level of MAO, while the group treated with HESP+EXCER had reduced MAO activity (Fig. 6), indicating the potential of combination therapy in the treatment of endometriosis and possible decrease of infertility. Our findings agree with the study of Campos *et al.* (2013) who reported the impact of exercise on MAO; an important source of ROS in the mitochondrial membrane (Campos *et al.,* 2013).

## CONCLUSION

This study ascertains that exercise improved the antioxidant status, while the combination of hesperidin and exercise have therapeutic effects in endometriosis.
